# Perchloride of Iron as an Escharotic

**Published:** 1862-02

**Authors:** 


Perchloride of Iron as an Escharotic.—*Messrs. Editors*:—It is often difficult to remove the fungous growth arising from excessive granulations after the suppuration of carbuncles, etc. Recently having a troublesome case of this kind, after using the usual remedies, such as nitrate of silver, sulphate of copper, without any benefit, I applied the perchloride of iron with complete success.

This powerful styptic will be found an excellent application in similar cases, and judging from its effects, it might be used with good results in removing the remains of polypus after extraction with the forceps, in bleeding piles, and in ulceration of the uterus.

I was led to make a trial of it in the case referred to, from a notice in a late number of the Reporter, of its application in cases of ingrowing toe-nail. A. Shiland.

The above, from the Medical and Surgical Reporter, suggests the idea that perchloride of iron may be employed upon the same principle, in the treatment of diseases of the gums, such as ulceration, sloughing, purient growth, etc. We have not yet tried it, but intend to do so, but now mention it, that others may try it; and we hope those who do so will report to us. T.

